# Congenital Adrenal Hyperplasia and Ehlers-Danlos Syndrome

**DOI:** 10.3389/fendo.2022.803226

**Published:** 2022-02-25

**Authors:** Roxana Marino, Angélica Moresco, Natalia Perez Garrido, Pablo Ramirez, Alicia Belgorosky

**Affiliations:** ^1^ Molecular Biology Laboratory, Endocrinology Service, Hospital de Pediatría Prof. Dr. Juan P. Garrahan, Buenos Aires, Argentina; ^2^ Genetics Service, Hospital de Pediatría Prof. Dr. Juan P. Garrahan, Buenos Aires, Argentina; ^3^ Endocrinology Service, Hospital de Pediatría Prof. Dr. Juan P. Garrahan, Buenos Aires, Argentina; ^4^ Consejo Nacional de Investigaciones Científicas y Técnicas (CONICET), Buenos Aires, Argentina

**Keywords:** congenital adrenal hyperplasia, CAH-X, *CYP21A2*, *TNXB*, Ehlers-Danlos Syndrome

## Abstract

Congenital adrenal hyperplasia (CAH) secondary to 21-hydroxylase deficiency is an autosomal recessive disorder. The 21-hydroxylase enzyme P450c21 is encoded by the *CYP21A2* gene located on chromosome 6p21.33 within the HLA major histocompatibility complex. This locus also contains the *CYP21A1P*, a non-functional pseudogene, that is highly homologous to the *CYP21A2* gene. Other duplicated genes are *C4A* and *C4B*, that encode two isoforms of complement factor C4, the *RP1* gene that encodes a serine/threonine protein kinase, and the *TNXB* gene that, encodes the extracellular matrix glycoprotein tenascin-X (TNX). TNX plays a role in collagen deposition by dermal fibroblasts and is expressed in the dermis of the skin and the connective tissue of the heart and skeletal muscle. During meiosis, misalignment may occur producing large gene deletions or gene conversion events resulting in chimeric genes. Chimeric recombination may occur between *TNXB* and *TNXA.* Three *TNXA/TNXB* chimeras have been described that differ in the junction site (CH1 to CH3) and result in a contiguous *CYP21A2* and *TNXB* gene deletion, causing CAH-X syndrome. *TNXB* deficiency is associated with Ehlers Danlos syndrome (EDS). EDS comprises a clinically and genetically heterogeneous group of connective tissue disorders. As molecular analysis of the *TNXB* gene is challenging, the TNX-deficient type EDS is probably underdiagnosed. In this minireview, we will address the different strategies of molecular analysis of the *TNXB*-gene, as well as copy number variations and genetic status of *TNXB* in different cohorts. Furthermore, clinical features of EDS and clinical recommendations for long-term follow-up are discussed.

## Introduction

Congenital adrenal hyperplasia (CAH) comprises a group of autosomal recessive enzymatic disorders, caused by a deficiency of one of the enzymes required for cortisol biosynthesis in the adrenal cortex. CAH is mostly associated with pathogenic variants in the 21-hydroxylase (*CYP21A2*) gene (1, 2). Residual enzyme activity defines the clinical severity of the disease. The most common form of CAH is 21-hydroxylase deficiency (21-OHD) accounting for 95% of cases. Prevalence of the most severe or classic forms is 1:16,000 live births in the Caucasian population, while the non-classic or late-onset form is the most common, with a prevalence between 1:1000-1:500 live births depending on ethnicity and geographic area (1–3).

**Figure 1:**
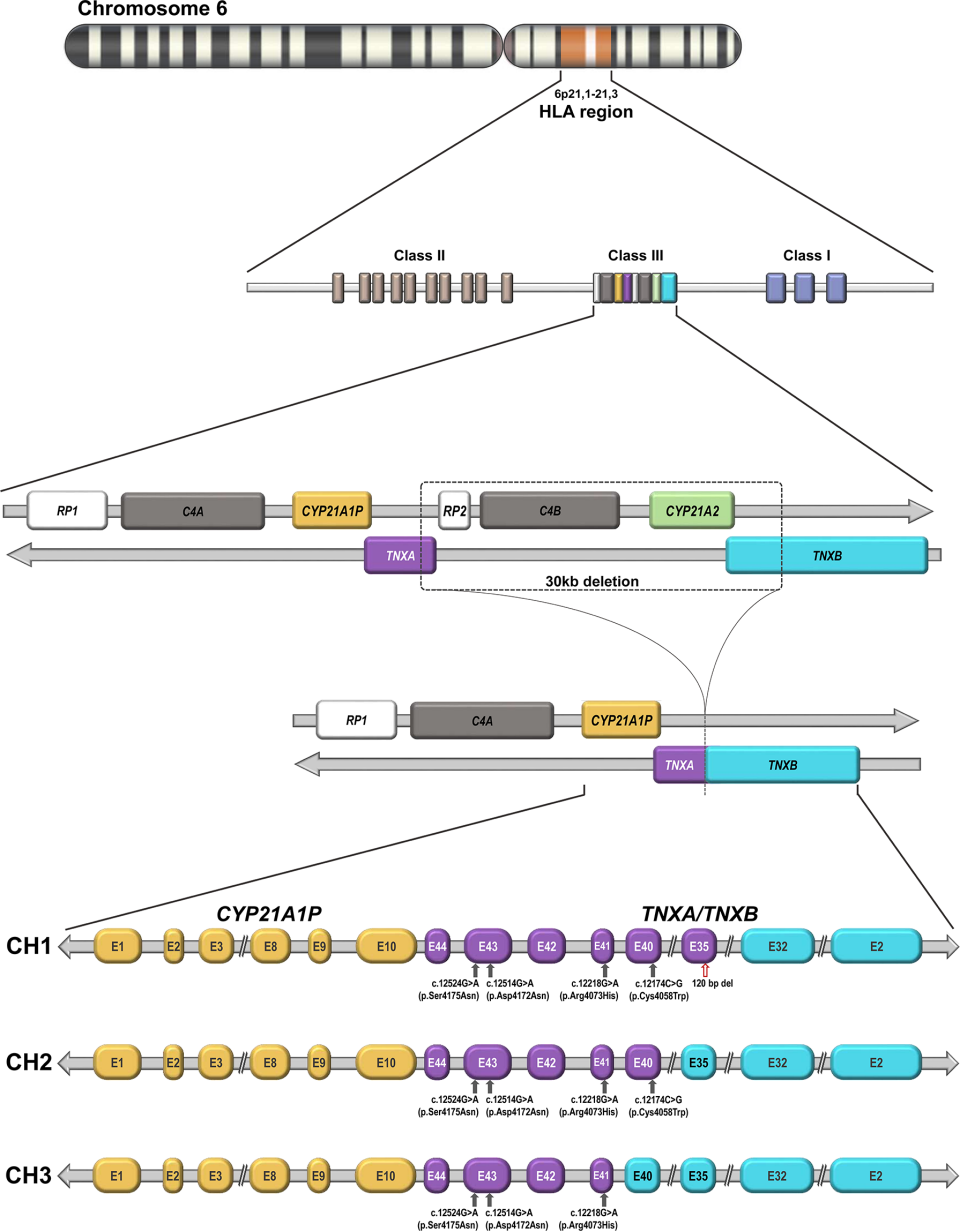
Genomic organization of the CYP21 locus on chromosome 6p21.1-21.3: The functional *CYP21A2* gene and its non-functional *CYP21A1P* pseudogene are arranged in tandem repeat with the two *C4* genes that encode factor four of the complement system, the serine–threonine nuclear protein kinase active gene *RP1* (*STK19*) and a truncated pseudogene *RP2* (*STK19P*). The functional *TNXB* and the *TNXA* pseudogene are located on the complement strand. Schematic representation of a 30-kb deletion as a result of unequal crossover during meiosis and the formation of the three described *TNXA/TNXB* chimeras (CH1, CH2 and CH3), which differ in the junction site. CH1 is characterized by a 120-bp deletion in exon and intron 35 carried over from *TNXA* pseudogene sequences. CH2 lacks this deletion but contains a pseudogene-derived variant c.12174C>G (p.Cys4058Trp) in *TNXB* exon 40. CH3 is characterized by the presence of any variant of a cluster of three pseudogene-derived variants (exon 41: c.12218G>A, p.Arg4073His; exon 43: c.12514G>A, p.Asp4172Asn and c.12524G>A, p.Ser4175Asn). These three pseudogene-derived variants do not always co-segregate in the three chimeras. *TNXB* gene exons are represented in cyan and *TNXA* gene exons in purple. The red arrow indicates a 120-bp deletion in exon 35 and grey arrows indicate different pseudogene-derived variants. CH1: *TNXA/TNXB* chimera 1. CH2: *TNXA/TNXB* chimera 2. CH3: *TNXA/TNXB* chimera 3.

The *CYP21A2* gene is located on the long arm of chromosome 6, within the major human histocompatibility complex (HLA), a region with a complex gene organization (4–7) (**Figure 1**). There is a nonfunctional pseudogene (C*YP21A1P)*, located approximately 30 kb from the *CYP21A2* gene. Both the functional gene and the pseudogene comprise ten exons that share a 98% nucleotide sequence identity (4–7). The close proximity and high level of homology between the functional and the non-functional gene facilitates misalignment resulting in recombination events that frequently produce large *CYP21A2* gene deletions and conversions as well as point pathogenic variants in the *CYP21A2* gene. The locus, is one of the most complex in the human genome since it contains three other genes, *RP1* also called *STK19* that encodes a serine/threonine protein kinase, *C4A* and *C4B* that encode two isoforms of complement factor C4, and *TNXB* that encodes an extracellular matrix glycoprotein tenascin-X (TNX), as well as two pseudogenes, *RP2* and *TNXA*, that together constitute a 30-kb genetic unit called the RCCX module (4–7). The genetic diversity of the RCCX module is highly attributable to nonallelic homologous recombination (NAHR). Unequal crossover during meiosis generates large structural rearrangements and copy number changes, whereas gene conversion mediates relatively short sequence transfers (8).

The *TNXA* and *TNXB* genes lie on the opposite strand of DNA from the other genes of the cluster and therefore have the opposite transcriptional direction. The last exon of *TNXA* and *TNXB* partially overlap the 3′ untranslated region of exon 10 in *CYP21A1P* and *CYP21A2*, respectively (9). *TNXB* is a large gene composed of 44 exons spanning 68.2 kb, whereas *TNXA* is a truncated gene of 4.5kb, homologous to exons 32 to 44 of *TNXB.*

As mentioned above, chimeric *CYP21A1P/CYP21A2* genes are caused by homologous recombination between *CYP21A2* and its pseudogene *CYP21A1P* as a result of unequal crossover and are found in 20-25% of alleles in CAH due to 21-OHD. To date, nine different *CYP21A1P/CYP21A2* genes have been characterized (10).

The unequal crossover may, in some cases, produce *TNXA/TNXB* chimeras from which the *CYP21A2* gene is completely removed (**Figure 1**). At present three different *TNXA/TNXB* chimeras have been described- CH1, CH2 and CH3-that differ in the junction site. CH1 is characterized by a 120-bp deletion in exon and intron 35 carried over from *TNXA* pseudogene sequences leading to haploinsufficiency (11). CH2 lacks this deletion but contains a pseudogene-derived variant- c.12174C>G (p.Cys4058Trp)- in *TNXB* exon 40 producing the loss of a critical disulfide bond in the tertiary structure of the TNX C-terminal fibrinogen-like domain (12). CH3 is characterized by the presence of any variant of a cluster of three pseudogene-derived variants -exon 41: c.12218G>A, (p.Arg4073His); exon 43: c.12514G>A (p.Asp4172Asn) and c.12524G>A (p.Ser4175Asn)-. The cluster of these three pseudogene-derived variants may differ in the haplotypes found in the three chimeras and they do not always co-segregate. Modeling and energy calculations suggest that the p.Arg4073His variant is detrimental to proper TNX folding while the remaining variants in the cluster did not significantly affect the folding energies in the models (13) . In addition, some CH1 haplotypes that harbor the 120-bp deletion in exon 35 but lack the p.Cys4058Trp variant in exon 40 have been found. This may be explained by the fact that the allele frequency of pseudogene derived-variants is not 100 percent. Since CH2 and CH3 produce altered proteins rather than reducing TNX expression, they are associated with a dominant-negative effect. The TNX protein belongs to a family of evolutionarily conserved large glycoproteins of the extracellular matrix. It plays a role in collagen deposition by dermal fibroblasts and is expressed in the dermis of the skin and in the connective tissue of the heart and skeletal muscle. *TNXB* deficiency leads to Ehlers-Danlos Syndrome (EDS) and up to 10% of CAH patients also have EDS, an entity called CAH-X. EDS comprises a clinically and genetically heterogeneous group of connective tissue disorders characterized by joint hypermobility, skin hyperextensibility, and tissue fragility (14).

As molecular analysis of the *TNXB* gene is challenging, the TNX-deficient type EDS is probably underdiagnosed. In this minireview, we will address the different strategies of molecular analysis of the *TNXB* gene, as well as copy number variations and genetic status of *TNXB* in different cohorts. Furthermore, clinical features of EDS and clinical recommendations for long-term follow-up are discussed.

## Molecular Analysis of the *TNXB* Gene

The first report of TNX deficiency was a description of a patient with CAH and EDS (15). In 2001, Schalkwijk et al. reported a subtype of EDS that is now known as classic-like EDS (clEDS) (16). clEDS is an autosomal recessive form of EDS and is caused by a deficiency of TNX encoded by the *TNXB* gene. The authors evaluated 151 patients with the classic hypermobility or vascular types of EDS, together with 168 patients with other conditions (psoriasis and rheumatoid arthritis) and 21 healthy individuals for the presence of TNX and tenascin-C by enzyme-linked immunosorbent assay. The patients were tested for the 30-kb deletion leading to a *TNXA/TNXB* chimeric gene by allele-specific PCR and for other point mutations by sequencing the coding sequence of the *TNXB* gene. Four of five TNX–deficient patients were identified to have homozygous *TNXB* mutations. Subsequently, the same authors reported an association between haploinsufficiency of the *TNXB* gene and the hypermobility-type EDS (hEDS) in 20 heterozygous family members; however, generalized joint hypermobility (GJH) was observed in only nine female patients (17).

The first evaluation of the potential clinical implications of *TNXB* heterozygosity in CAH patients was reported by Merke et al. in a large prospective observational study from the National Institutes of Health (NIH) (11). One hundred ninety-three unrelated patients with CAH were evaluated clinically for manifestations of EDS and genetically for *TNXB* mutations. DNA was analyzed for the presence of a contiguous gene deletion syndrome caused by deletion of *CYP21A2* and its flanking gene *TNXB* by PCR multiplex ligation-dependent probe amplification (MLPA) and confirmed by Southern blot. This deletion generated a *TNXA/TNXB* chimera characterized by a 120-bp deletion in exon 35 of the *TNXB* gene, which is replaced by *TNXA* sequences. In addition, *TNXB* sequencing was performed in a group of patients with one or more joint or skin manifestations. Heterozygosity for the *TNXB* deletion was observed in 7% of CAH patients, who were considered to have CAH-X syndrome. Here, the association between the hypermobility phenotype and *TNXB*-gene haploinsufficiency was established. In 2015, the same authors identified a pseudogene-derived variant- c.12174C>G (p.C4058W)- representing a novel *TNXA/TNXB* chimera that did not involve a 120-bp deletion in exon 35 in seven families with CAH-X (12). Interestingly, this variant did not affect the protein expression of tenascin in dermal fibroblasts and for this missense variant a dominant-negative mechanism was proposed, which is different from the haploinsufficiency caused by the above-described chimera. Of 246 CAH probands screened, 14 carried previously described *TNXA/TNXB* chimeras (CH1) while seven unrelated patients carried the novel *TNXB* variant (CH2) resulting in a prevalence of CAH-X of 8.5% (12). The same authors later reported three patients with biallelic CAH-X and identified a novel dominant-negative chimera (CH3) characterized by any of three *TNXB* variants [exon 41: c.12218G>A (p.Arg4073His); 191 exon 43: c.12514G>A (p.Asp4172Asn) and c.12524G>A (p.Ser4175Asn)] (**Figure 1**). This study presented evidence for disrupted TNX function, since by computational data the p.Arg4073His variant was predicted to reduce protein-folding energy by interfering with a cation-pi interaction between p.Arg4073 and p.Phe4080 (13).

Molecular analysis of the *TNXB* gene is challenging due to the presence of the pseudogene, which makes next-generation sequencing highly complicated in these cases. In 2019, Lao et al. reported a high-throughput CAH-X screening method based on allele-specific PCR to assess the copy numbers of *TNXB* exons 35 and 40. The method is compatible with either quantitative PCR or droplet digital PCR and allows detection of CH1 and CH2. Using this methodology, the authors found a 15.6% prevalence of CAH-X, which was higher than previously estimated. The prevalence was especially high (29.2%) in subjects with a 30-kb deletion genotype (18).

In 2020, Gao et al. assessed the prevalence of the chimeric *TNXA/TNXB* gene and clinical symptoms in a Chinese cohort of 424 21-OHD patients. MLPA analysis and Sanger sequencing was performed to genetically identify the CAH-X syndrome. In this cohort, 14% of the patients with 21-OHD were found to have a chimeric *TNXA*/*TNXB* gene (19). Finally, also in 2020, our group reported the molecular *TNXB*-gene status and clinical evaluation of the EDS phenotype in a cohort of 337 Argentine 21-OHD patients to assess the prevalence of this condition in our population. *TNXB* gene analysis was performed in 66 unrelated CAH patients that were carriers of the 30-kb *CYP21A2* gene deletion. A molecular strategy based on MLPA and Sanger sequencing analysis was developed for the detection of the three previously described *TNXA/TNXB* chimeras (20). The overall prevalence of CAH-X in 21-OHD patients in our cohort was 14%, which was similar to that previously found in the large cohort from the NIH and in the Chinese population (15% and 14%, respectively). In our population of 21-OHD patients carrying the 30-kb *CYP21A2* gene deletion in which the junction site was downstream exon 7 both in the homozygous or the heterozygous state, the incidence of *TNXA/TNXB* chimeras was 73% (48/66), similar to the prevalence of 62.8% (59/94) found in the Chinese population. On the other hand, in the NIH cohort a prevalence of 29.2% (21/72) was reported. The reason for the lower prevalence found in the latter study is that the authors reported the presence of *TNXA/TNXB* chimeras in CAH patients that were carriers of all types of 30-kb *CYP21A2* deletions described.

In addition to *TNXA/TNXB* chimeras, pathogenic variants in the *TNXB* gene have been described as a less frequent cause of TNX deficiency. Pathogenic variants were detected in the coding region of the EGF-like repeats, the fibronectin type III domain or C-terminal domain structurally related to fibrinogen of the TNX protein. Moreover, recently a splice donor site variant has been described as a cause of the hEDS type (21). Finally, the variable prevalence of CAH-X reported in the different cohorts might be related to the molecular strategies used.

## Clinical Manifestations of CAH-X Patients

CAH-X patients are reported to have a wide range of connective tissue abnormalities, including generalized joint hypermobility, subluxations, chronic arthralgias, soft or velvety skin, mild skin hyperextensibility, and variable systemic manifestations. The severity of the phenotype may be correlated with the dosage of the dominant alleles, as monoallelic CAH-X is associated with hEDS, the mildest and most common EDS type, and biallelic CAH-X with the more severe clEDS subtype.

Biallelic CAH-X patients resemble the clEDS type, with extreme joint laxity, with or without recurrent joint dislocations, and skin hyperextensibility with a velvety skin texture and absence of atrophic scarring. Easily bruisable skin and soft-tissue injuries as well as organ prolapse, pes planus, piezogenic papules, chronic pain, arthralgias, and cardiac abnormalities have been described. Thus far, 12 patients with biallelic CAH-X syndrome have been reported (13, 15, 16, 19, 20); however, clinical information is not available for all cases. Our group reported four biallelic CAH-X patients (two with a CH1/CH1 and two with a CH1/CH2 combination) (20). Both CH1/CH2 patients had a more severe EDS phenotype, with greater skin involvement. Nevertheless, the low number of homozygous patients reported to date limits the possibility to draw robust conclusions based on these data. In addition, in the latter patients cardiac defects were detected; one had an atrial septal and the other a mild pulmonary valve defect. None of the patients developed either atrophic scarring, organ prolapse, or any other complications during the 3 years of follow-up; however, these observations are limited by the young age of our patients and the short-term follow-up. Chen et al. reported three biallelic patients of 14, 19, and 29 years of age, all displaying unique combinations of *TNXB* variants in both alleles; one of them was homozygous for CH2/CH2, the other was a CH2/CH3 compound, and the third a CH2/CH1 compound (13). All of them had skin hyperextensibility and significant joint hypermobility. Joint laxity was extreme and two patients had a history of joint dislocations, chronic arthralgias, and chronic tendinitis and/or bursitis. Unlike our findings, the authors reported widened atrophic scarring, rectal prolapse, severe gastroesophageal reflux, high palate, and elongated uvula in all three biallelic patients. Mild ventricular enlargement was detected in two patients. Currently, the limited number of cases reported and the heterogeneous combination of *TNXB* variants they display make it difficult to establish a certain genotype/phenotype correlation.

Long-term follow-up is needed to specifically evaluate quality of life in CAH-X patients. On the other hand, clEDS patients (without CAH) are affected by soft-tissue fragility and long-term complications (13, 15, 16, 19, 20).

Most patients with the monoallelic form of CAH-X syndrome present with the clinical spectrum of hEDS with variable expression at different stages of life, predominantly characterized by GJH, mild skin hyperextensibility, and soft velvety skin, without abnormal scarring. Related musculoskeletal complications, such as recurrent joint dislocations, pes planus, and chronic arthralgias, have been reported. Other associated features include functional gastrointestinal alterations and cardiac disorders. Although this type of EDS may cause chronic pain and reduced quality of life, life-threatening complications are uncommon (11, 12, 15, 17, 19, 20).

The underlying chimera translates into different degrees of hEDS phenotypes. Compared to *TNXB* haploinsufficiency caused by CAH-X CH1, a dominant negative effect related to CAH-X CH2 causes a more severe phenotype with increased joint and skin manifestations (12, 20). Gastrointestinal disorders, such as chronic gastroesophageal reflux and irritable bowel syndrome, hernias, and organ prolapse, are also more frequently reported in patients that are heterozygous for CAH-X CH2 than in those with CAH-X CH1. Data on the phenotype associated with the less frequent CAH-X CH3 are scarce (12, 19, 20, 22–24). Recently, Gao et al. reported the presence of CH3 in 11 patients; however, clinical information was available for only one of them, who had joint hypermobility and poor wound healing (19). In our cohort, CH3 was only found in one monoallelic patient who was not available for clinical evaluation (20).

Although the exact role of CAH-related hormonal factors and/or chronic glucocorticoid treatment in connective tissue dysplasia is not yet completely understood, it has been shown that CAH-X patients are consistently more severely affected than patients with homozygous or heterozygous TNX-deficient-type EDS without CAH (11, 12, 16, 19, 25).

Furthermore, a phenotype that is varied and usually milder than that of monoallelic CAH-X patients has been reported in the relatives of CAH-X patients, who were carriers of one CAH-X allele, but not affected by CAH. Some carriers were observed to have less joint, heart, or gastrointestinal symptoms, while others were asymptomatic (12, 13, 17, 19, 20). On the other hand, connective tissue dysplasia has been described in CAH patients without demonstrated *TNXB* deficiency (11, 12).

Recently, Lao et al. reported a novel cause of CAH-X syndrome, not associated with pathogenic *TNXA/TNXB* chimeras but due to a *TNXB* splice donor site variant (21). As currently *TNXB* testing remains challenging, at least in the routine diagnostic approach, the diagnosis of CAH-X still relies on clinical evaluation.

**Figure 2:**
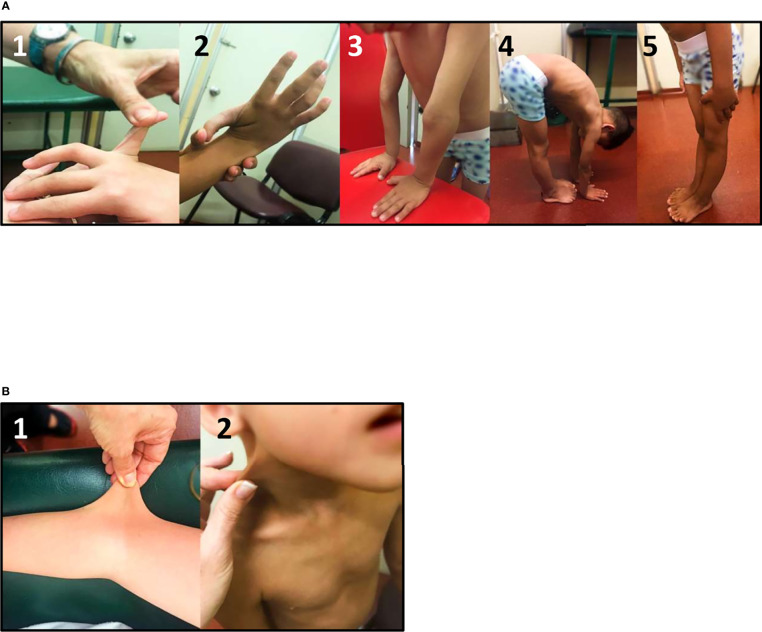
Clinical evaluation of CAH-X Syndrome patients: **(A)** Generalized joint hypermobility Beighton score: 1) fifth finger extension test, 2) wrist flexion thumb abduction test, 3) elbow extension test, 4) trunk and hip flexion test, and 5) knee extension test. **(B)** Skin extensibility can be measured by lifting the cutaneous and subcutaneous layers of the skin and is considered hyperextensible if it can be stretched over 1.5 cm at the forearm and the dorsum of hands, and 3 cm for neck, elbow, and knees. Photographs shown belong to a biallelic CAH-X patient (compound heterozygous for CH1 and CH2).

Connective tissue dysplasia should be evaluated in all CAH patients, especially in those harboring a deletion in the *CYP21A2* gene. Screening for GJH and other soft tissue features presents age-related difficulties and should therefore be adapted to age, systematically evaluated, and retrospectively asked for. The Beighton Score (shown in **Figure 2**) remains the most objective assessment tool to measure GJH (22, 26).

Knowledge of the *TNXB* status in CAH will not only offer patients a better understanding of their symptomatology with the burden of the diagnosis of a second genetic disease, but also assures specific clinical management with a focus on preventing musculoskeletal manifestations and complications. Cardiovascular alterations may be underreported in CAH-X patients, probably related to the difficulties patients without cardiac symptoms may have to access specific diagnostic studies, such as cardiac magnetic resonance imaging (MRI), outside the context of a research study protocol, as observed in different series. A variable prevalence of cardiac abnormalities was found in different studies using different diagnostic methods.

Currently, no specific medical or genetic therapies are available for CAH-X patients. Management consists of interdisciplinary medical care, rehabilitation, and monitoring of major and organ-specific complications (24, 27, 28). Further *TNXB* and CAH-X studies are necessary to define detailed surveillance guidelines for these and other long-term complications and to develop prevention strategies.

## Conclusions

Different studies on the *CYP21A2* gene have improved our knowledge on *TNXB*-related disorders. The *TNXB* gene encodes an extracellular matrix glycoprotein named TNX. EDS may be due to *TNXB* deficiency and up to 10% of 21-OHD CAH patients also have CAH-X. Chimeric recombination of the *TNXB* and *TNXA* genes may occur, and three *TNXA/TNXB* chimeras that differ in the junction site (CH1 to CH3) resulting in a contiguous *CYP21A2* and *TNXB* gene deletion, named CAH-X syndrome, have been described. Molecular studies are the gold standard to assess the presence of *TNXA/TNXB* chimeras. On the other hand, molecular analysis of the *TNXB* gene is challenging due to the presence of a pseudogene and next generation sequencing is highly complicated in these cases. For this reason, among others, TNX-deficient type EDS may be underdiagnosed. The variable prevalence of CAH-X reported in different cohorts may be related to the molecular strategies applied. Systematic study of *TNXB* status in individuals with a previous diagnosis of CAH and carriers of the complete 30-kb deletion of *CYP21A2* is highly recommended. Moreover, molecular genetic testing of CAH-21OHD should include *TNXA/TNXB* chimera analysis (29).

EDS comprises a clinically and genetically heterogeneous group of connective tissue disorders characterized by joint hypermobility, skin hyperextensibility, and tissue fragility as well as cardiovascular alterations. Cardiac disorders, in particular heart valve abnormalities, may be underdiagnosed, probably because of the young age of the majority of reported CAH-X patients and EDS-related cardiac abnormalities may appear with aging. In addition, it is unlikely that patients without heart symptoms are routinely checked for cardiac abnormalities with echocardiogram and/or cardiac MRI and currently only data from patients participating in specific CAH-X research studies are available. Severity of the phenotype may be correlated with the dosage of the dominant alleles, as monoallelic CAH-X patients have the mildest and most common EDS type and biallelic CAH-X patients the more severe clEDS subtype. CAH-X patients are consistently more severely affected than patients with homozygous or heterozygous TNX-deficient type EDS without CAH; however, the impact of the hormonal milieu on TNX and its role in connective-tissue pathophysiology is still poorly understood. In this line, carriers of one CAH-X allele who are not affected with CAH have a varied and milder EDS phenotype than monoallelic CAH-X patients.

Once the diagnosis of CAH-X has been established, it is advisable to guarantee long-term follow-up of these patients by medical specialists with a focus on preventing musculoskeletal manifestations and complications.

Finally, in order to prevent long-term musculoskeletal disorders, timely diagnosis of CAH-X is important and physical therapy for joint instability is recommended. In addition, molecular characterization of CAH-X is relevant for genetic counseling.

## Ethics Statement

Written informed consent was obtained from the participant’s legal guardians for the publication of any identifiable material in this study.

## Author Contributions

All authors contributed equally to design of the manuscript. RM, AM, and AB wrote the first draft of the manuscript. All authors contributed to manuscript revision, read, and approved the submitted version.

## Funding

Fondo Nacional de Ciencia y Tecnología, Argentina, Director of the Grant, Award number: 2016, 0028.

## Conflict of Interest

The authors declare that the research was conducted in the absence of any commercial or financial relationships that could be construed as a potential conflict of interest.

## Publisher’s Note

All claims expressed in this article are solely those of the authors and do not necessarily represent those of their affiliated organizations, or those of the publisher, the editors and the reviewers. Any product that may be evaluated in this article, or claim that may be made by its manufacturer, is not guaranteed or endorsed by the publisher.
